# Biofortification of *Chlorella vulgaris* With Selenium and Iron Microminerals and Investigation of Some Bioactive Compounds: A Promising Dietary Supplement

**DOI:** 10.1002/fsn3.72230

**Published:** 2026-09-06

**Authors:** Masoumeh Rezaei Moshaei, Mohammad Azadbakht, Akbar Hajizadeh Moghaddam, Mahbobeh Ghanbarzadeh

**Affiliations:** ^1^ Faculty of Biotechnology Amol University of Special Modern Technologies Amol Iran; ^2^ Department of Pharmacognosy and Biotechnology, Faculty of Pharmacy Mazandaran University of Medical Sciences Sari Iran; ^3^ Medicinal Plants Research Center Mazandaran University of Medical Sciences Sari Iran; ^4^ Department of Biology University of Mazandaran Babolsar Iran; ^5^ Department of Biotechnology of Microalgae Production and Processing Academic Center for Education, Culture & Research (ACECR) Mazandaran Iran

**Keywords:** antioxidant activity, bioaccumulation, *Chlorella vulgaris*, iron, photosynthesis pigments, selenium

## Abstract

Microalgae are excellent bioaccumulators of selenium (Se) and iron (Fe), making them ideal for use in the fortification of food and feed supplements. This study aimed to investigate the effects of varying concentrations of Se and Fe on the growth of *Chlorella vulgaris* and its capacity to accumulate these microminerals. The optimal concentration of each micronutrient was determined by evaluating growth parameters and metal accumulation, and the effects of these metals on various bioactive compounds were then analyzed. The highest specific growth rate and final biomass productivity were found in 50 mg L^−1^ Fe. The maximum accumulation of Se and Fe in *C. vulgaris* cells reached 81.54 ± 0.46 mg kg^−1^ DW at 45 mg L^−1^ Se and 26798.51 ± 1.49 mg kg^−1^ DW at 150 mg L^−1^ Fe, respectively. Chlorophyll a content increased in *C. vulgaris* treated with 15 mg L^−1^ Se and 50 mg L^−1^ Fe. In contrast, the contents of β‐carotene and lycopene were not significantly affected by Se and Fe treatments. Total flavonoid content and antioxidant activity significantly increased in 15 mg L^−1^ Se and 50 mg L^−1^ Fe. The fatty acid profile indicated that unsaturated fatty acids, particularly linoleic, alpha‐linolenic, and palmitoleic acid, were the dominant components identified under Se and Fe treatments. This study suggested that *C. vulgaris* enriched with optimal levels of Se and Fe can be regarded as a promising candidate for the development of functional foods and nutritional purposes, due to its rich bioactive compounds.

## Graphical abstract

This study was designed to investigate the effects of varying concentrations of selenium and iron on the growth of *Chlorella vulgaris* microalgae and its potential for bioaccumulation of these essential micronutrients. The optimal concentration for each metal was determined through detailed assessment of growth parameters. Key bioactive compounds—phenolics, flavonoids, chlorophyll, lycopene, β‐carotene, and soluble protein—were measured alongside antioxidant activity (DPPH and FRAP). Results identified optimal doses for enhancing both nutritional value and antioxidant capacity of this microalga.

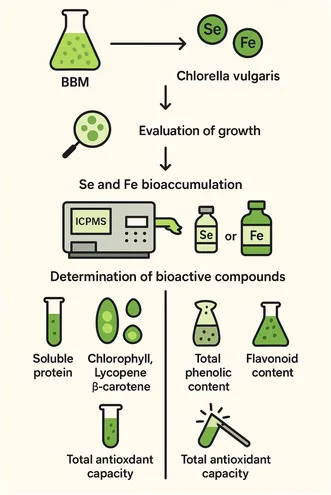

## 1 Introduction

Mineral and vitamin deficiencies are frequently referred to as “hidden hunger” or “micronutrient deficiencies,” which is currently recognized as one of the most serious challenges to human health (Lowe 2021). Selenium (Se) and iron (Fe) deficiencies are among the most well‐known forms of micronutrient malnutrition in humans. Fe deficiency is the most prevalent nutritional disorder, particularly affecting children, adolescent girls, and pregnant women (Kumar et al. 2022). The gastrointestinal side effects associated with iron sulfate supplements, along with the requirement for prolonged treatment (up to 6 months), have rendered the issue of Fe deficiency and its resultant anemia a persistent challenge (Iolascon et al. 2024). The main cause of these deficiencies is a lack of Fe‐rich foods in the diet or the body's inability to absorb Fe for various reasons (Rusu et al. 2020).

Se deficiency is another significant concern, as it can lead to psychological issues such as depression, apathy, anxiety, and aggression. This micronutrient plays a vital role in maintaining proper thyroid function, supporting heart health, reducing the risk of cancer and neurodegenerative diseases, boosting the immune system, and safeguarding the body from free radicals when present in appropriate amounts (Lanza and Dos Reis 2021). Nevertheless, an excess of these compounds promotes the creation of ROS, leading to oxidative stress and eventual cell demise (X. Sun et al. 2014). According to the Institute of Medicine, the Recommended Dietary Allowance (RDA) for selenium in adults is 55 μg day^−1^, with a tolerable upper intake level (UL) of 400 μg day^−1^. For iron, the RDA is 8 mg day^−1^ for adult men and 18 mg day^−1^ for adult women of reproductive age, with a tolerable upper intake level of 45 mg day^−1^ (Medicine, I. o 2000, 2001). Supplementing with inorganic Se is important and sensitive because it can easily reach toxic concentrations. Studies have shown that microalgae can convert inorganic Se into organic form (Zhang et al. 2024).

Major intervention strategies against micronutrient malnutrition include supplementation for vulnerable groups, improving dietary habits, and biofortifying commonly consumed foods with micronutrients (Haridas et al. 2022). Biofortification is an economical and sustainable approach to addressing micronutrient deficiencies. Research has demonstrated that this method can greatly enhance nutrient consumption (Ghanbarzadeh et al. 2024). Dietary supplements derived from natural sources have become popular due to their lack of side effects, low cost, and antioxidant properties. In this context, microalgae are excellent sources of a wide and diverse range of essential compounds needed by the body, including carbohydrates, amino acids, unsaturated fatty acids, fibers, vitamins, and minerals (Torres‐Tiji et al. 2020). They are also rich in high antioxidant compounds and are recognized as scavengers of free radicals. Due to their high chlorophyll and fiber content, microalgae consumption can prevent the absorption of heavy metals and other toxic substances entering the body through the digestive system, while also facilitating their excretion (Islam et al. 2017). Studies have shown that microalgae‐based supplements can meet a broader range of needs in smaller volumes compared to plant‐based supplements (Siddiqui et al. 2024). Among these, *Chlorella* and *Spirulina* species have gained greater popularity in the global market.

*Chlorella vulgaris* ( *C. vulgaris* ) is a spherical, single‐celled microalgae that lives in water (Safi et al. 2014). The cell wall of *Chlorella* can absorb and eliminate heavy metals and chemical toxins, thereby strengthening the immune system. It has also been shown that *Chlorella* contains the highest amount of chlorophyll among all plant species, contributing to its role as a natural antioxidant and metabolic regulator. This microalgae compensates for potential deficiencies in the diet by providing fiber, amino acids, and minerals due to its rich and bioactive compounds (Janczyk et al. 2007; Safi et al. 2014). Additionally, it is a suitable supplement for the elderly due to its calcium and essential vitamins (Bito et al. 2020). Fortified *C. vulgaris* is a green microalgae grown in a nutrient‐rich environment containing essential micronutrients and minerals. Given the global problems caused by mineral deficiencies, biofortification can be a promising strategy to combat its adverse consequences (Chomchan et al. 2017). Therefore, the present study aims to biofortify *C. vulgaris* with essential micronutrients, including Fe and Se. Additionally, it seeks to investigate the biochemical composition of the enriched microalgae to facilitate the development of functional and nutraceutical dietary supplements.

## 2 Materials and Methods

### 2.1 Cultivation Conditions and Media Content of *C. vulgaris*

*C. vulgaris* (with the accession number 0 M991707.1 in the NCBI database) was received from ACECR's microalgae culture collection, Iran. This microalgae was cultured in Bold's Basal Medium (BBM) in sterile and specific conditions to optimize its growth and productivity. To prepare the inoculum, *C. vulgaris* was cultivated and preserved in sterilized Erlenmeyer flasks filled with BBM, under incubation conditions of 25°C ± 0.5°C, a pH range of 7 to 7.5, and a photoperiod cycle of 16:8 h (light: dark), at a light intensity of 120 μmol photons m^−2^ s^−1^. Oxygen levels in the culture were maintained through continuous aeration at 0.5 vvm using an air pump. The ideal temperature for growth was 25°C to 30°C. These conditions maximized biomass production and ensured the healthy growth of *C. vulgaris* in the BBM medium.

The components of basal culture medium (HC 1963) are as follows: Macronutrients (KH_2_PO_4_ 175 mg L^−1^, K_2_HPO_4_ 75 mg L^−1^, MgSO_4_.7H_2_O 75 mg L^−1^, CaCl_2_. 2H_2_O 25 mg L^−1^, NaNO_3_ 250 mg L^−1^, NaCl 25 mg L^−1^), Alkaline EDTA Solution (KOH 31 mg L^−1^, EDTA 50 mg L^−1^), Acidified Fe Solution (FeSO_4_.7H_2_O 4.98 mg L^−1^, H_2_SO_4_ 1 μL), Boron Solution (H_3_BO_3_ 11.42 mg L^−^1), Trace Metal Solution (ZnSO_4_.7H_2_O 8.82 mg L^−1^, MnCl_2_.4H_2_O 1.44 mg L^−1^, MoO_3_ 0.71 mg L^−1^, CuSO_4_. 5H_2_O 1.57 mg L^−1^ Co (NO_3_)_2_. 6H_2_O 0.49 mg L^−1^). It should be noted that the basal concentration of FeSO₄ (4.98 mg L^−1^) inherent to the BBM formulation was maintained across all treatment groups, including the control. The tested Fe concentrations (50, 100, and 150 mg L^−1^) were supplemented in addition to this basal level.

### 2.2 The Design of the Experiment

To investigate how sodium selenite (Na_2_SeO_3_) and ferrous sulfate (FeSO_4_) influence the growth of *C. vulgaris* and its capacity to accumulate these micronutrients, *C. vulgaris* was grown in the BBM with different concentrations of Se (0, 15, 30, and 45 mg L^−1^) and Fe (0, 50, 100, and 150 mg L^−1^). Cultures grown in the absence of sodium selenite and ferrous sulfate served as the control groups. In the preliminary experiments, the optimal Se and Fe concentrations for enriching *C. vulgaris* with these micronutrients were determined by assessing metal accumulation, growth parameters, Recommended Dietary Allowances (RDAs) for Se and Fe intake (Siva Kiran et al. 2015; Trumbo et al. 2001), as well as the daily dosage of *C. vulgaris* consumption (Siva Kiran et al. 2015). After determining the optimal concentration, we conducted a series of experiments to cultivate *C. vulgaris* under these ideal conditions. All experiments were conducted in triplicate (*n* = 3) to ensure reproducibility. We used three biological replicates to analyze various phytochemicals, including photosynthetic pigments, phenolic and flavonoid content, fatty acid composition, and antioxidant activity in *C. vulgaris* .

### 2.3 Growth Analysis

#### 2.3.1 Evaluation of Growth, Specific Growth Rate, and Biomass Productivity

The growth of *C. vulgaris* was evaluated using the method described by Leganés et al. (1987) which involved measuring the optical density (OD) at 680 nm every two days throughout the experiment with a spectrophotometer (i3 UV–VIS, Hanon Instruments, Jinan, China). This method is commonly used to monitor microalgae growth, as the OD correlates with biomass concentration.

The specific growth rate (μ) was calculated from the change in OD over time using the following Equation (1):

(1) $$\mu =\frac{\left(\mathrm{Ln}\;N_{2}-\mathrm{Ln}\;N_{1}\right)}{\left(t_{2}-t_{1}\right)}$$

where: $\;N_{2}$ is the OD at the time $t_{2}$, $N_{1}$ is the OD at the initial time ($t_{1}$), and $t_{2}-t_{1}$ is the time interval. This equation allows researchers to quantify the growth dynamics of the culture over the experimental period.

The calculation of biomass productivity was performed using Equation (2), where X_2_ represents the biomass concentration (dry weight measurement) at time t_2_, and X_1_ denotes the biomass concentration (dry weight measurement) at time t_1_. The formula for biomass productivity is given by:

(2) $$\text{Biomass productivity}=\frac{\left(X2-X1\right)}{\left(t2-t1\right)}$$

This equation essentially calculates the change in biomass over a specific time interval, providing insight into how effectively biomass is being produced.

### 2.4 Determination of se and Fe Bioaccumulation

The Se and Fe concentration in the lyophilized biomass of *C. vulgaris* was assessed using an inductively coupled plasma mass spectrometer (ICP‐MS). Initially, 0.2 g of lyophilized sample was mixed with 5 mL of nitric acid (HNO_3_) at 180°C for 2 h to break down the cellular structure and release the trace elements into solution. Following the digestion process, the sample was cooled to room temperature (RT) and subsequently brought to 10 mL with ultrapure water to achieve a suitable concentration for analysis, filtered through Whatman filter paper to remove any particulate matter that could interfere with the analysis, and then the prepared solutions were introduced into the ICP‐MS system for precise measurement of total concentrations of Se and Fe (Zdziebłowska et al. 2024).

### 2.5 Determination of Soluble Protein

The soluble protein concentration was determined using the Bradford dye‐binding assay, which relies on the interaction between Coomassie Brilliant Blue G‐250 and protein molecules (Bradford 1976). Bovine serum albumin (BSA) was used as a standard reference. To prepare the protein reagent, 100 mg of Coomassie Brilliant Blue G‐250 was completely solubilized in 95% ethanol (50 mL). The solution was then supplemented with 100 mL of 85% (w v^−1^) phosphoric acid. The final mixture was diluted to a total volume of 1 L, resulting in a reagent with concentrations of 8.5% (w v^−1^) phosphoric acid, 0.01% (w v^−1^) Coomassie Brilliant Blue G‐250, and 4.7% (w v^−1^) ethanol. For the protein assay, 100 μL of Tris–HCl extract from *C. vulgaris* containing 10 to 100 μg of protein and each test tube received 5 mL of the protein reagent, followed by vortex mixing of the contents. Measurements of absorbance at 595 nm were performed initially at 2 min and subsequently before the 1‐h mark, with a reagent blank prepared from 100 μL of Tris–HCl buffer and 5 mL of protein reagent.

### 2.6 Determination of Photosynthetic Pigments

#### 2.6.1 Chlorophyll

Chlorophyll content was measured after incubating samples in 99% methanol at 4°C for 24 h. The pigment isolation process from microalgae cells involves several steps. First, 0.015 g of dried biomass of *C. vulgaris* was suspended in 3 mL of absolute methanol and vortexed for 1 min. Next, the suspension was heated in a water bath at 60°C for 30 min, allowed to cool at RT, and centrifuged at 10,000 rpm for 3 min. The absorbance of the pigment extract was measured at 665 and 652 nm for chlorophyll (a + b) using a UV–VIS spectrophotometer (Hanon). The chlorophyll concentration was calculated in micrograms per milliliter according to the method of Porra et al. (1989) (Porra et al. 1989).

#### 2.6.2 Lycopene and β‐Carotene

All pigments for lycopene and β‐carotene measurement in dried biomass of *C. vulgaris* were extracted with acetone and hexane in a 4:6 ratio. Following centrifugation (8000 rpm, 10 min), the OD of the supernatant was measured at wavelengths of 453, 505, 645, and 663 nm by a UV–VIS spectrophotometer (Hanon) (Nagata and Yamashita 1992). The concentrations of lycopene and β‐carotene were estimated using proposed equations (Formulas 1 and 2).

(1) $$\begin{array}{c} \text{Lycopene}\;\left(\mathrm{mg}\;{\mathrm{mL}}^{-1}\right)=-0.0458A_{663}+0.20{\text{4A}}_{645}\\ \;+0.37{\text{2A}}_{505}–0.0806A_{453} \end{array}$$

(2) $$\begin{array}{c} \beta -\text{carotene}\;\left(\mathrm{mg}\;{\mathrm{mL}}^{-1}\right)=0.21{\text{6A}}_{663}–1.22A_{645}\\ \;–0.30{\text{4A}}_{505}+0.45{\text{2A}}_{453} \end{array}$$

### 2.7 Quantification of Total Phenolic Compounds

The total phenolic content was estimated according to the Folin–Ciocalteu method (Nićiforović et al. 2010). This technique utilizes the Folin–Ciocalteu reagent to measure total phenols by oxidizing phenolates into a blue complex, which is quantified at a wavelength of 756 nm. Gallic acid was used as a standard reagent. To determine the total phenolic content, a reaction mixture was prepared by combining 0.5 mL of extract with 2.5 mL of 10% (v v^−1^) Folin–Ciocalteu reagent and 2 mL of 7.5% (w v^−1^) sodium carbonate solution, and the samples were allowed to stand at RT for 60 min. Then, the absorbance against a reagent blank was measured at 765 nm by a UV–VIS spectrophotometer (Hanon). The results are reported as milligrams of gallic acid equivalent per gram of extract (mg GAE g^−1^ extract).

### 2.8 Determination of Flavonoids

The aluminium chloride colorimetric method was utilized to measure flavonoid concentrations (Ghanbarzadeh et al. 2022). Quercetin was used as the standard for creating the calibration curve. For the flavonoid content analysis, 0.5 mL of ethanolic extract from *C. vulgaris* was mixed with 0.1 mL of 10% aluminium chloride dissolved in methanol, 0.1 mL of 1 M potassium acetate, and 2.8 mL of distilled water. Following a 30‐min incubation period at RT, absorbance was recorded at 415 nm using a UV–VIS spectrophotometer (Hanon, China). The 10% aluminium chloride with an equal volume of distilled water was used for the blank.

### 2.9 Estimation of Fatty Acid Methyl Ester (FAME) Content

Biomass was gathered and dried using a freeze‐drier in the direct transesterification method for analyzing fatty acid methyl esters (FAME). The lipids from 0.1 g of freeze‐dried biomass of *C. vulgaris* were hydrolyzed and methyl‐esterified with 300 μL of 2% H_2_SO_4_ and methanol solution at 80°C for 2 h (Duong et al. 2015). After esterification and cooling to RT, 300 μL each of 0.9% NaCl (w/v) and hexane were added and vortexed for 20 s. Centrifugation (16,000 × g, 3 min) achieved phase separation, allowing the hexane layer, which contained the fatty acid methyl esters (FAMEs), to be used for FAME profile characterization via gas chromatography analysis. The experimental conditions were adapted from Guan et al. (2011) (Guan et al. 2011).

### 2.10 Determination of DPPH Radical Scavenging Activity

The DPPH (2,2‐Diphenyl‐1‐picrylhydrazyl) assay was employed to assess free radical‐scavenging activity. This method relies on reducing DPPH in a methanol solution when a hydrogen‐donating antioxidant is present, forming a non‐radical species from DPPH‐H. Initially, 0.5 mL of methanolic extracts (80 μg/mL) from microalgae was added to a methanolic solution of DPPH at an equal concentration. Then, the resulting mixture was shaken and stored in the dark. After incubating for 30 min at RT, the absorbance was measured at 517 nm (Blois 1958). To ensure the accuracy and reproducibility of the results, all measurements were conducted in three independent biological replicates and three intra‐assay replicates using three distinct spectrophotometers, all of which produced consistent and comparable results. The percentage of inhibition for the free radical scavenging capacity was determined by applying the following equation:

(3) $$\text{Inhibition}\;\left(\%\right)=\frac{A0-\mathrm{As}}{A0}\times 100$$

where: A_0_ represents the absorbance of the control, and A_S_ denotes the absorbance of the sample after incubation.

### 2.11 Determination of Ferric‐Reducing Antioxidant Power (FRAP)

Antioxidant activity was also evaluated using the FRAP assay as originally described by Benzie and Strain (1996) (Benzie and Strain 1996). 100 μL of sample was combined with 3 mL of freshly prepared FRAP reagent (containing 10 mM TPTZ in 40 mM HCl, 20 mM FeCl₃.6H_2_O, and 300 mM acetate buffer, pH 3.6; mixed 1:1:10 v/v/v). Following a 5‐min incubation period at 37°C, the absorbance was recorded at 593 nm. A standard curve was prepared using 10 mM ferrous sulfate (FeSO_4_.7H_2_O). The activity was expressed in mM Fe^2+^.

### 2.12 Statistical Analysis

The experiment was designed as a completely randomized design (CRD) and performed in at least three replicates. Graphs and figures were generated using GraphPad Prism 9. Results are expressed as mean ± SD. The data were compared using a one‐way analysis of variance (ANOVA) followed by Duncan's test. The data analyses were performed using SPSS 26 software. All *p‐values* < 0.05 were considered statistically significant.

## 3 Results

### 3.1 The Impact of Varying se Concentrations on the Specific Growth Rate (SGR), Final Biomass Productivity, and Bioaccumulation

Based on the results, the addition of sodium selenite (Na_2_SeO_3_) to the microalgae nutrient medium showed that the effects of Se treatments on the growth parameters of *C. vulgaris* were not statistically significant compared with the control group (*p* > 0.05) (Table 1). Meanwhile, at supraoptimal Se concentrations (≥ 75 mg L^−1^), the culture medium exhibited a distinct reddish discoloration, accompanied by a significant reduction in biomass productivity and specific growth rate, ultimately resulting in cell death. These effects are attributed to selenium toxicity at elevated doses, which induces oxidative stress and disrupts essential metabolic processes in *C. vulgaris* (data not shown). It was also found that the Se content in *C. vulgaris* directly depends on its concentration in the culture medium and increased significantly with the increase of Se concentration from 15 to 45 mg L^−1^. As the Se accumulation is shown in Table 2, the maximum accumulation was about 81.54 ± 0.46 mg kg^−1^ DW in *C. vulgaris* treated with 45 mg L^−1^, while the minimum accumulation of this element was obtained in the control group.

**TABLE 1:** Effects of different Se concentrations on the specific growth rate (SGR), biomass productivity, and dry weight (DW) in *C. vulgaris* .

Treatment	SGR (d^−1^)	Biomass productivity (mg^−1^ mL^−1^d^−1^)	DW (mg mL^−1^)
Control	0.074 ± 0.001^a^	0.145 ± 0.003^a^	3.76 ± 0.055^a^
15 mg L^−1^ Na_2_SeO_3_	0.074 ± 0.002^a^	0.145 ± 0.008^a^	3.76 ± 0.156^a^
30 mg L^−1^ Na_2_SeO_3_	0.075 ± 0.001^a^	0.150 ± 0.005^a^	3.84 ± 0.110^a^
45 mg L^−1^ Na_2_SeO_3_	0.076 ± 0.000^a^	0.152 ± 0.001^a^	3.90 ± 0.18^a^

*Note:* In each column, means with different letters indicate significant differences at *p*‐value < 0.05.

**TABLE 2:** Accumulation of Se in *C. vulgaris* .

Treatment	Accumulation (mg kg^−1^ DW)
Control	23.5 ± 1^d^
15 mg L^−1^ Na_2_SeO_3_	51.84 ± 0.66^c^
30 mg L^−1^ Na_2_SeO_3_	69.14 ± 0.86^b^
45 mg L^−1^ Na_2_SeO_3_	81.54 ± 0.46^a^

*Note:* In each column, means with different letters indicate significant differences at *p*‐value < 0.05.

### 3.2 Effects of Different Concentrations of Fe on the SGR, Final Biomass Productivity, and Bioaccumulation

The addition of Fe to the microalgae nutrient medium significantly increased the SGR, biomass productivity, and DW in *C. vulgaris* fortified with different concentrations of Fe (50, 100, 150 mg L^−1^), which were compared with the control sample (Table 3). Although the lowest SGR, biomass productivity, and DW levels were recorded at 1.31 ± 0.075 d^−1^, 0.186 ± 0.014 mg^−1^ mL^−1^ d^−1^, and 3.720 ± 0.217 mg mL^−1^ in the control sample, respectively, no significant difference was observed between different concentrations of Fe in these parameters (Table 3). Further, it was found that the Fe content in *C. vulgaris* directly depends on its concentration in the culture medium. As the Fe accumulation is shown in Table 4, the maximum accumulation was about 26798.51 ± 1.49 mg kg^−1^ DW in *C. vulgaris* fortified with 150 mg L^−1^, while the minimum accumulation of this element was obtained in the control group.

**TABLE 3:** Effects of different concentrations of Fe on the specific growth rate (SGR), biomass productivity, and dry weight (DW) in *C. vulgaris* .

Treatment	SGR (d^−1^)	Biomass productivity (mg^−1^ mL^−1^d^−1^)	DW (mg mL^−1^)
Control	1.31 ± 0.075^a^	0.186 ± 0.014^a^	3.720 ± 0.217^a^
50 mg L^−1^ FeSo_4_	1.47 ± 0.054^b^	0.227 ± 0.012^b^	4.380 ± 0.192^b^
100 mg L^−1^ FeSo_4_	1.47 ± 0.059^b^	0.226 ± 0.017^b^	4.360 ± 0.275^b^
150 mg L^−1^FeSo_4_	1.47 ± 0.058^b^	0.227 ± 0.020^b^	4.371 ± 0.315^b^

*Note:* In each column, means with different letters indicate significant differences at *p*‐value < 0.05.

**TABLE 4:** Accumulation of Fe in *C. vulgaris* .

Treatment	Accumulation (mg kg^−1^ DW)
Control	818.43 ± 0.57^d^
50 mg L^−1^ FeSo_4_	5303.31 ± 2.69^c^
100 mg L^−1^ FeSo_4_	11324.34 ± 1.34^b^
150 mg L^−1^FeSo_4_	26798.51 ± 1.49^a^

*Note:* In each column, means with different letters indicate significant differences at *p*‐value < 0.05.

### 3.3 Effect of Selected Concentrations of se (15 mg L^−1^) and Fe (50 mg L^−1^) on Some Phytochemicals in *C. vulgaris*

**FIGURE 1:**
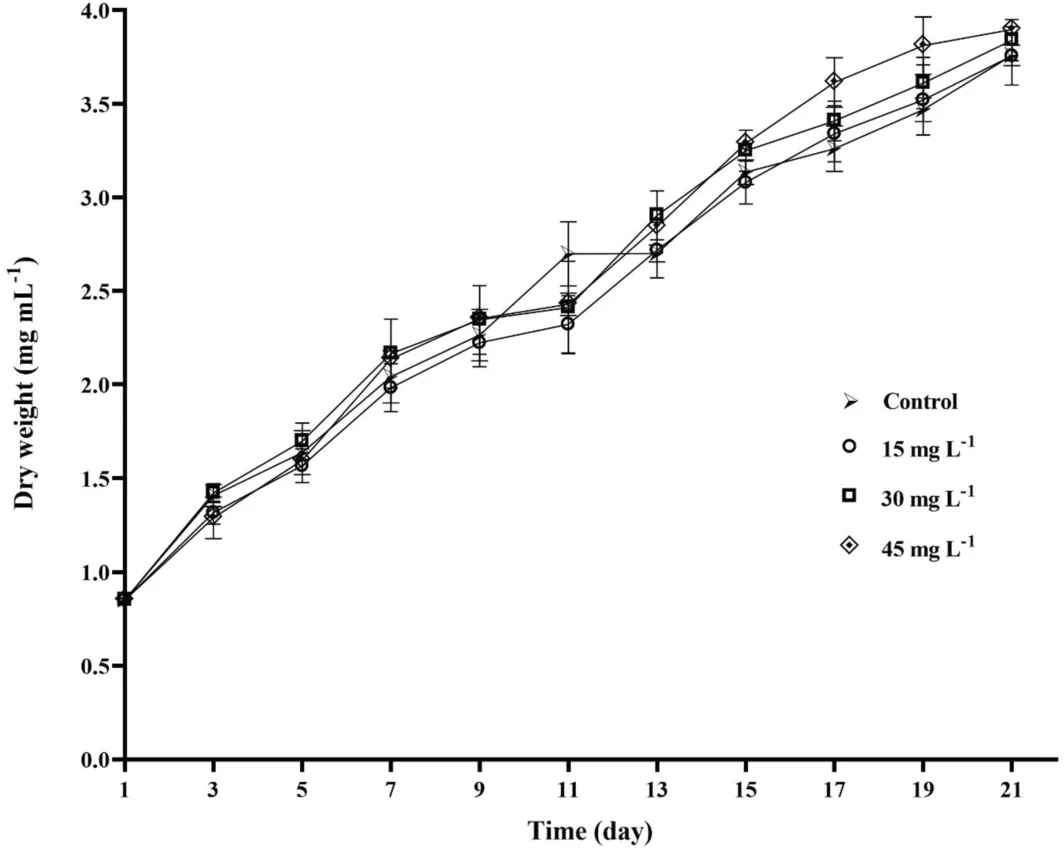
Growth curves (biomass in dry weight) of *C. vulgaris* at different concentrations of Na_2_SeO_3_ over the time (days) of the experiment.

**FIGURE 2:**
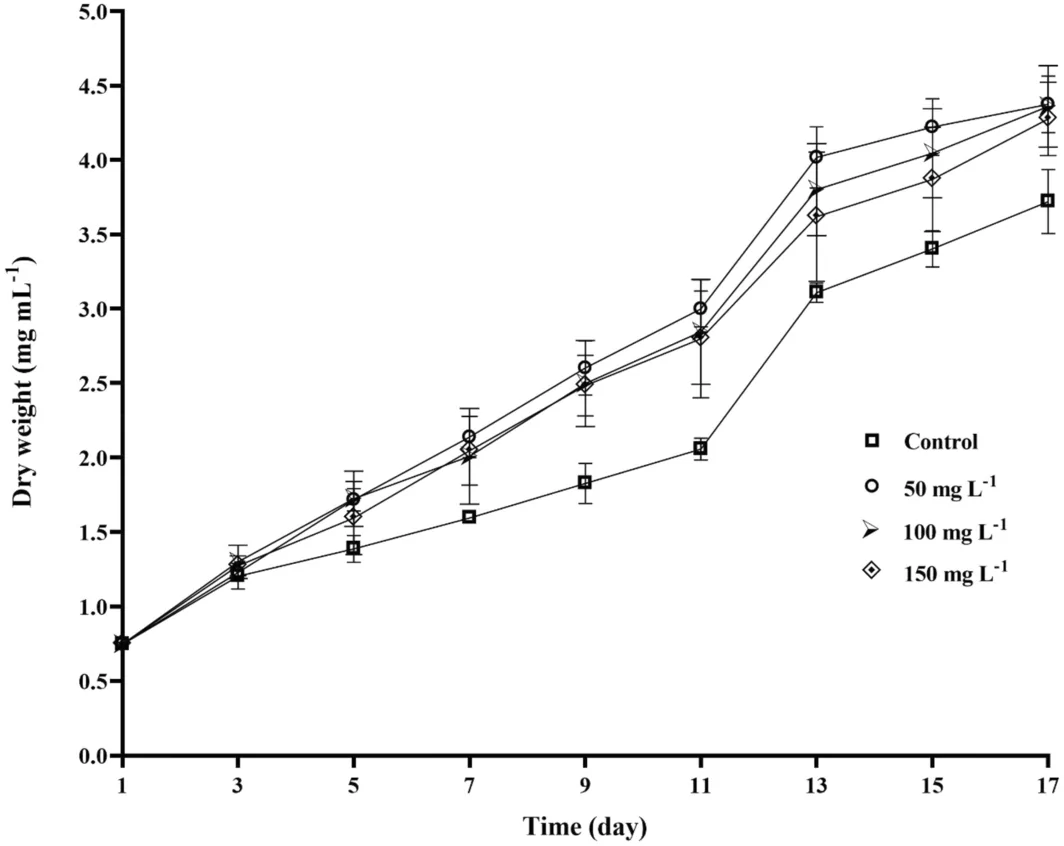
Growth curves (biomass in dry weight) of *C. vulgaris* at different concentrations of FeSO_4_ over the time (days) of the experiment.

A concentration of 15 mg L^−1^ of Se and 50 mg L^−1^ of Fe was selected based on several factors: growth parameters (as shown in Figures 1 and 2, Tables 1 and 3), Se and Fe accumulation in the cells of *C. vulgaris* that as shown in Tables 2 and 4 respectively. The recommended daily consumption of *C. vulgaris* for a healthy adult is 3–10 g day^−1^ (C. A. Wang et al. 2024), and the required adult daily dose for Se and Fe consumption containing 50–70 μg day^−1^ and 8–18 mg day^−1^, respectively (Trumbo et al. 2001). To investigate the effectiveness of biofortification, the effect of selected concentrations of Se (15 mg L^−1^) and Fe (50 mg L^−1^) on some phytochemicals in *C. vulgaris* was evaluated and compared with the control (Table 5). The results showed that chlorophyll a, total phenol, and total flavonoids contents were significantly higher in *C. vulgaris* treated with Na_2_SeO_3_ (15 mg L^−1^) compared with the control, reaching 626.82 ± 3.3, 14.18 ± 1.48, and 18.20 ± 0.41 mg g^−1^ dry weight, respectively (vs. 454.59 ± 0.31, 10.30 ± 0.44, and 3.29 ± 0.94 mg g^−1^ dry weight in the control) (Table 5). Similarly, β‐carotene and lycopene contents were significantly higher in *C. vulgaris* treated with Fe (50 mg L^−1^) compared with the control, reaching 10.39 ± 0.46 and 8.66 ± 0.42 μg mg^−1^ dry weight, respectively (vs. 10.04 ± 0.31 and 7.97 ± 0.17 μg mg^−1^ dry weight in the control) (Table 5). In contrast, chlorophyll b and soluble protein contents were significantly higher in the control sample (679.58 ± 1.30 μg mg^−1^ dry weight and 8.174 ± 1.33 mg g^−1^ dry weight, respectively) compared with the Se‐ and Fe‐treated groups (Table 5).

**TABLE 5:** Effect of Se and Fe on some phytochemicals in fortified *C. vulgaris*.

Parameters	Control	15 mg L^−1^ Na_2_SeO_3_	50 mg L^−1^ FeSo_4_
Chlorophyll *a* (μg mg^−1^ dry weight)	454.59 ± 0.31^c^	626.82 ± 3.34^a^	510.87 ± 0.28^b^
Chlorophyll *b* (μg mg^−1^ dry weight)	679.58 ± 1.30^a^	591.67 ± 11.55^b^	468.16 ± 0.28^c^
Chlorophyll (a+b) (μg mg^−1^ dry weight)	1134.17 ± 1.47^b^	1218.49 ± 13.66^a^	979.03 ± 0.74^c^
β‐carotene (μg mg^−1^ dry weight)	10.04 ± 0.31^ab^	9.57 ± 0.38^b^	10.39 ± 0.46^a^
Lycopene (μg mg^−1^ dry weight)	7.97 ± 0.17^ab^	7.71 ± 0.56^b^	8.66 ± 0.42^a^
Soluble protein (mg g^−1^ dry weight)	8.174 ± 1.33^a^	3.634 ± 0.26^b^	3.539 ± 0.70^b^
Total phenolics (mg g^−1^ dry weight)	10.30 ± 0.44^b^	14.18 ± 1.48^a^	9.88 ± 0.07^b^
Total flavonoids (mg g^−1^ dry weight)	3.29 ± 0.94^c^	18.20 ± 0.41^a^	8.98 ± 1.70^b^

*Note:* In each column, means with different letters indicate significant differences at *p*‐value < 0.05.

### 3.4 Antioxidant Activity in Biofortified *C. vulgaris*

**FIGURE 3:**
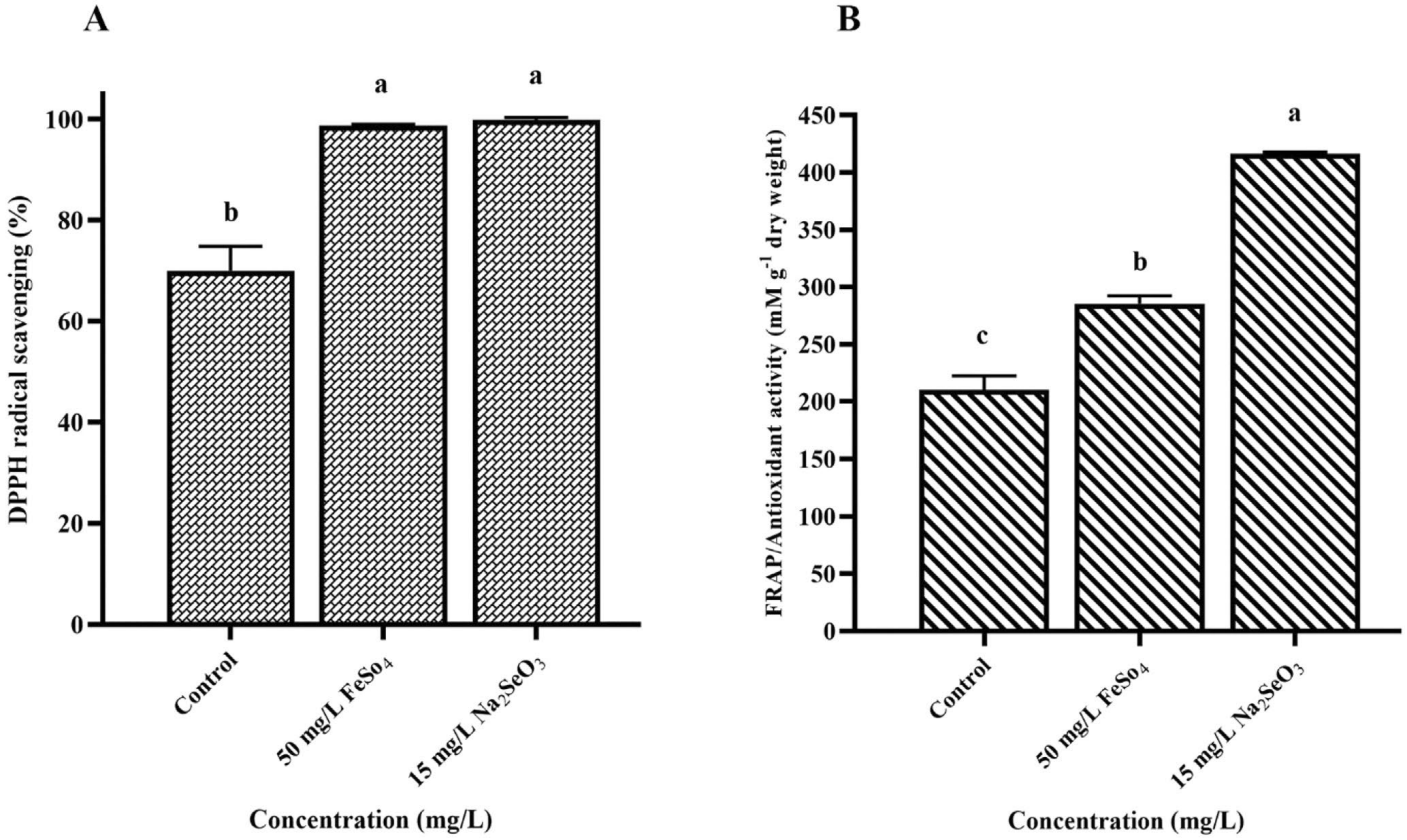
Antioxidant activity of fortified *C. vulgaris* by the DPPH radical scavenging (A) and FRAP method (B). (Columns with different letters indicate significant differences at *p*‐value < 0.05).

The antioxidant activity of *C. vulgaris* biofortified with optimal Se and Fe concentrations was evaluated by the FRAP assay and DPPH free radical scavenging method. The results showed that DPPH scavenging was significantly higher in *C. vulgaris* fortified with Na_2_SeO_3_ (15 mg L^−1^) and FeSO_4_ (50 mg L^−1^) compared with the control, reaching 99.86 ± 0.005 and 98.65 ± 0.004%, respectively (vs. 70.01% in the control) (Figure 3A). Likewise, the FRAP assay showed significantly higher antioxidant activity in *C. vulgaris* fortified with Na_2_SeO_3_ compared with the control, reaching 416.19 ± 1.48 mM g^−1^ dry weight (vs. 210.19 ± 12.21 mM g^−1^ dry weight in the control) (Figure 3B). Figure 3 shows the antioxidant activity of fortified *C. vulgaris* using the FRAP assay and the DPPH radical scavenging method.

### 3.5 Fatty Acid Composition in Biofortified *C. vulgaris*

**FIGURE 4:**
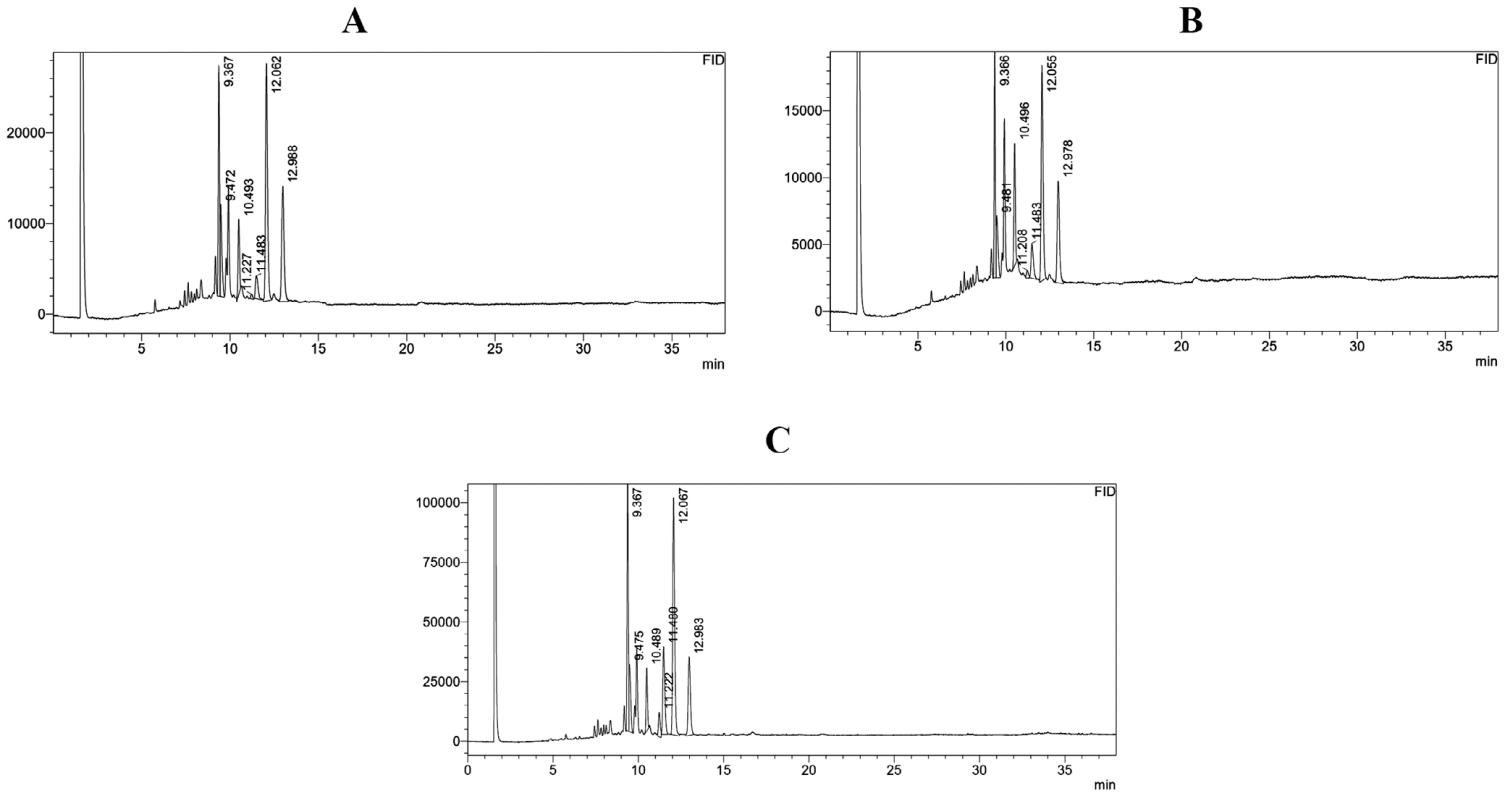
Fatty acids composition in *C. vulgaris* fortified with Fe (A), Se (B), and control sample (C).

The composition of essential fatty acids in *C. vulgaris* treated with Se and Fe showed that linoleic acid, α‐Linolenic acid, and palmitoleic acid were significantly higher in the group treated with 50 mg L^−1^ Fe compared with the control, reaching 35.266 ± 0.31, 19.767 ± 0.25, and 11.106 ± 0.12%, respectively (vs. 33.48 ± 0.20%, 12.76 ± 0.24%, and 7.63 ± 0.40% in the control). Heptadecenoic acid content was also significantly higher in *C. vulgaris* fortified with 15 mg L^−1^ Na_2_SeO_3_ compared with the control, reaching 13.360 ± 0.36% (vs. 6.46 ± 0.30% in the control) (Table 6, Figure 4). In contrast, oleic acid and stearic acid contents were significantly higher in the control sample (12.80 ± 0.28% and 3.13 ± 0.03%, respectively) compared with the Se‐ and Fe‐treated groups (Table 6, Figure 4).

**TABLE 6:** Fatty acid composition (%) of *C. vulgaris* fortified with Se and Fe, compared with the control.

Fatty acids	Structure	Control (%)	15 mg L^−1^ Na_2_SeO_3_ (%)	50 mg L^−1^ FeSo_4_ (%)
Linoleic acid (C18:2 n‐6)	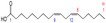	33.48 ± 0.20^b^	31.95 ± 0.41^c^	35.26 ± 0.31^a^
Palmitic acid (C16:0)	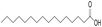	23.72 ± 0.26^a^	20.49 ± 0.51^b^	20.81 ± 0.31^b^
α‐Linolenic acid (C18:3 n‐3)	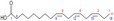	12.76 ± 0.24^c^	18.23 ± 0.34^b^	19.76 ± 0.25^a^
Oleic acid (C18:1 n‐9)	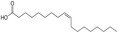	12.80 ± 0.28^a^	6.18 ± 0.06^b^	4.33 ± 0.2^c^
Palmitoleic acid (C16:1)	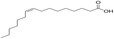	7.63 ± 0.40^c^	8.60 ± 0.38^b^	11.10 ± 0.12^a^
Heptadecenoic acid (C17:1)	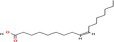	6.46 ± 0.30^c^	13.36 ± 0.36^a^	8.06 ± 0.14^b^
Stearic acid (C18:0)	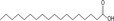	3.13 ± 0.03^a^	1.16 ± 0.18^b^	0.64 ± 0.04^c^

*Note:* In each column, means with different letters indicate significant differences at *p*‐value < 0.05.

## 4 Discussion

In the present study, the impact of selenium (Se) and iron (Fe) levels on the growth, bioaccumulation, antioxidant activity, fatty acid profile, and phytochemical content of *C. vulgaris* was investigated. Selenium (Se) is an essential micronutrient for microalgae, aiding in protein and lipid synthesis and providing oxidative protection (Araie and Shiraiwa 2016; Ekelund and Danilov 2001; Schiavon et al. 2017). However, its role is dose‐dependent; at low concentrations it promotes growth, but at high levels it becomes toxic and inhibits growth (Doušková et al. 2007; X. Sun et al. 2014).

This study found that *C. vulgaris* microalgae growth was stimulated at sodium selenite concentrations between 15 and 45 mg L^−1^, demonstrating tolerance to Se. Growth promotion is linked to enhanced oxidase activity (Zhao et al. 2019). However, concentrations exceeding 45 mg L^−1^ inhibited growth and produced a red precipitate of Se in the medium, resulting from the algae's conversion of oxidized Se (Babaei et al. 2017). Additionally, high levels of Se may inhibit photosynthesis, alter cell ultrastructure, reduce metabolic processes, decrease growth rate, and potentially cause cell damage and death (Babaei et al. 2017; Schiavon et al. 2017; Vítová et al. 2011). Gojkovic et al. (2014) reported that *Chlorella sorokiniana* can physiologically adapt to a Se concentration of 40 mg L^−1^ by accumulating significant amounts of intracellular Se in the form of SeMet and other Se‐containing amino acids due to its reductive metabolism (Gojkovic et al. 2013, 2014). These results are consistent with the study of Zhong and Cheng (2017), Gojkovic et al. (2015), and Sun et al. (2014), who found that low selenite levels stimulate *Chlorella* growth, while high Se concentrations significantly inhibit it and reduce biomass (Gojkovic et al. 2015; X. Sun et al. 2014; Zhong and Cheng 2017).

Fe is an essential micronutrient for algal growth, supporting key processes like photosynthesis and respiration (Abidizadegan et al. 2023; Rizwan et al. 2017). In this study, while growth improved with added Fe, no significant differences were found in key metrics (such as growth rate and biomass) between 50 and 150 mg L^−1^ of FeSO_4_. Therefore, the lowest concentration (50 mg L^−1^) was sufficient for optimal growth of *C. vulgaris* . These findings are in agreement with the observations made by Iriani et al. (2011), who reported that *Chlorella* sp. cultured at various Fe levels exhibited optimal growth at the lowest Fe level (Dian Iriani et al. 2011). Isani et al. (2022) and Ismaiel et al. (2018) indicated that there was no significant difference in the growth of *Arthrospira platensis* when cultivated at 1–10 mg L^−1^ levels of Fe (Isani et al. 2022; Ismaiel et al. 2018). Others reported that increased Fe improved growth rates in several species, including *C. vulgaris* , and enhanced its biomass productivity (Liu et al. 2008; Pádrová et al. 2015; Rastar et al. 2018; Weng et al. 2007). Conversely, Fe deficiency is known to reduce photosynthetic capacity and slow growth (Rizwan et al. 2017).

The results also revealed that *C. vulgaris* cells can absorb and accumulate Se and Fe. This process is facilitated by the presence of various metal‐binding groups (such as carboxyl, amino, and phosphate groups) on the microalgal cell wall, which attract metal ions and enable their passage through the cell membrane (Arunakumara and Zhang 2008; Ayed et al. 2015; J. Wang and Chen 2006). The *C. vulgaris* successfully accumulated Se and Fe when grown in solutions containing up to 45 mg L^−1^ Na_2_SeO_3_ and 150 mg L^−1^ FeSO_4_, respectively. Cell Se content increased up to 3.5‐fold with higher Se levels (Table 2), while Fe content rose by up to 32.8‐fold with more Fe (Table 4). This enrichment was achieved without reducing biomass, showing *C. vulgaris* has high tolerance and is an effective bioaccumulator of both Se and Fe.

The accumulation of Se and Fe in *C. vulgaris* at the concentrations identified in this study has important implications for human health. Se is essential for antioxidant defense, thyroid hormone metabolism, and immune function. Since Se in microalgae predominantly occurs in organic forms such as selenomethionine and selenocysteine, which exhibit higher bioavailability and lower toxicity than inorganic forms, Se‐enriched *C. vulgaris* represents a potentially promising dietary source worthy of further investigation (Schiavon et al. 2017). However, excessive Se intake can lead to selenosis, characterized by hair loss, gastrointestinal disturbances, and neurological damage (Vinceti et al. 2014), highlighting the importance of dosage control in supplement applications. Similarly, Fe‐enriched *C. vulgaris* may represent a valuable plant‐based iron source, particularly for populations at risk of deficiency. However, excessively high Fe levels may promote oxidative stress via the Fenton reaction, potentially contributing to liver and cardiovascular damage (Yan et al. 2022). In this regard, 50 mg L^−1^ Fe is more appropriate for dietary use, as it supports optimal biomass quality and bioactive compound content without the risks associated with excess Fe accumulation. Future studies should evaluate the bioaccessibility and bioavailability of Se and Fe, and the safety of *C. vulgaris* using in vivo models and clinical trials before formally establishing its application as a functional food or dietary supplement.

Se bioaccumulation in microalgae is a strategy for both detoxification and environmental adaptation (Schiavon et al. 2017). General defense mechanisms against heavy metals include producing metal‐binding proteins, using transporters to exclude metals, and sequestering or excreting them. For algae specifically, tolerance is largely determined by their capacity to prevent oxidative damage, secrete chelators, actively pump out ions, and limit uptake (Pinto et al. 2003).

Soluble protein in *C. vulgaris* decreased significantly—by over half—when exposed to 15 mg L^−1^ Se or 50 mg L^−1^ Fe compared with the control (Table 5). This occurs because heavy metal ions disrupt the protein environment, causing misfolding, impairing DNA repair, affecting membranes, and ultimately disturbing protein function (Hasan et al. 2017). Iriani et al. (2011) reported the highest protein content (8.34 mg g^−1^ dry weight) at the lowest Fe concentration (0.35 mg L^−1^). In contrast, Iriani et al. (2017) observed the lowest protein content (4.86 mg g^−1^ dry weight) in *Chlorella* sp. treated with 1.68 mg L^−1^ Fe^3+^ after 21 days of cultivation (D Iriani et al. 2017).

Supplementation with Se and Fe enhanced photosynthesis in *C. vulgaris* , as reflected by a significant increase in chlorophyll a content at 15 mg L^−1^ Se and 50 mg L^−1^ Fe compared with the control, whereas β‐carotene and lycopene contents were not significantly affected by either treatment (Table 5). The significant rise in chlorophyll‐*a* at 15 mg L^−1^ Se is attributed to Se's influence on chloroplast function and photosynthetic efficiency, including its interaction with the electron transport chain and its role in improving Fe absorption (Zhong and Cheng 2017). At low concentrations, Se positively regulates enzymes involved in chlorophyll biosynthesis, thereby stimulating pigment production (Saffaryazdi et al. 2012).

Fe is essential for photosynthesis, chlorophyll synthesis, and chloroplast function, while Se at optimal levels acts as a cofactor in the electron transport chain (Roosta et al. 2018). At 50 mg L^−1^ Fe, chlorophyll‐*a* content increased significantly compared with the control, whereas β‐carotene and lycopene contents showed no significant change (Table 5). The rise in chlorophyll‐*a* at this Fe level is likely due to Fe's role in porphyrin synthesis and chlorophyll structure (Shetty and Miller 1966).

Lycopene and beta‐carotene are the most common carotenoids produced by plants. Microalgae also produce carotenoids as essential pigments and efficient antioxidants that protect them from oxidative damage. Carotenoids are crucial for several processes in photosynthesis and photoprotection, including the absorption of light and the transfer of energy, and also protect the photosynthetic apparatus against damage from intense light, particularly from reactive oxygen species (ROS) such as singlet oxygen and other free radicals (T. Sun et al. 2022). Carotenoids, due to their antioxidant properties, play a crucial role in protecting photosynthetic membranes from light oxidation and neutralizing peroxide radicals. Thus, the levels of carotenoids can influence changes in chlorophyll content by preventing the degradation of chlorophyll and lipid membranes in chloroplasts, ultimately leading to an increase in the number of green pigments in cells (Zhong and Cheng 2017). Although Ghanbarzadeh et al. (2022) reported a significant increase in β‐carotene content under Fe treatment, no significant change in β‐carotene or lycopene content was observed in the present study following Se or Fe supplementation (Table 5). Nevertheless, the significant increase in chlorophyll‐*a* content indicates that an appropriate concentration of Se and Fe enhances photosynthetic activity in *C. vulgaris* . Phenolics are major phytochemicals responsible for antioxidant activity in plants and microalgae, with bioactive roles including metal chelation, radical scavenging, and disease risk reduction (Liang et al. 2010; Rahaiee et al. 2025). In this study, total phenolic and flavonoid contents were significantly higher in *C. vulgaris* treated with 15 mg L^−1^ Se compared with the control (Table 5), possibly due to enhanced sugar accumulation or Se‐induced activation of phenylpropanoid pathway enzymes such as phenylalanine ammonia‐lyase (Lei et al. 2014). Flavonoid content was also significantly higher at 50 mg L^−1^ Fe compared with the control, whereas total phenolic content showed no significant difference between the Fe treatment and the control (Table 5). The elevated polyphenol levels under Se and Fe treatments are likely a stress response to ROS, stimulating antioxidant production, and are consistent with previous reports (Dey and Raychaudhuri 2023; Lattanzio 2013). These experimental outcomes correlate well with the results previously published by Ghanbarzadeh et al. (2022).

The antioxidant activity of the methanolic extract from *C. vulgaris* was determined by DPPH radical scavenging and the FRAP method. The results indicated that *C. vulgaris* treated with 15 mg L^−1^ of Se and 50 mg L^−1^ of Fe exhibited a more significant scavenging effect on DPPH than the control group (Figure 3). The treatment with Se and Fe promotes the accumulation of various phytochemicals, including phenolic compounds, known for their antioxidant properties. These compounds play a crucial role in scavenging free radicals, thereby increasing the overall antioxidant capacity of the microalgae (Abdel‐Karim et al. 2019). Data analysis demonstrated significant interdependence of Se and Fe concentrations and the levels of pigments, total phenolics, total flavonoids, and fatty acids. Additionally, we observed a connection between radical inhibition and the content of these metabolites in enriched *C. vulgaris* . The strong correlation indicates that Se and Fe enhance the synthesis of the mentioned metabolites and reduce free radicals. This may suggest that these metabolites are effective at scavenging free radicals, highlighting their potential health and wellness benefits (Gąsecka et al. 2015; Takyar et al. 2019). Other research has shown that phenolics contribute to antioxidant activity; however, the scavenging rate is not exclusively related to total phenolic or flavonoid content. This lack of correlation may be due to the varying antioxidant activities of different phenolic compounds or groups (Palacios et al. 2011).

Fatty acid biosynthesis in microalgae is not fully understood and is strongly influenced by growth stage and environmental conditions. In this study, *C. vulgaris* treated with 15 mg L^−1^ Na_2_SeO₃ showed a significant increase in unsaturated fatty acids, including α‐linolenic, palmitoleic, and heptadecenoic acids, compared with the control (Table 6). These increases are attributed to Se‐induced modulation of metabolic pathways and stress responses that stimulate fatty acid synthesis (Zhong and Cheng 2017). Similarly, Fe treatment enhanced unsaturated fatty acid production due to Fe's essential role in lipid‐related metabolic processes.

Fe enhances fatty acid biosynthesis by acting as an enzymatic cofactor, promoting growth, lipid accumulation, and increased production of unsaturated fatty acids in *Chlorella* (Liu et al. 2008). Polyunsaturated fatty acids such as linoleic and α‐linolenic acids play key structural roles, while saturated and monounsaturated fatty acids function mainly as storage lipids. In this study, Se treatment decreased oleic acid and increased linolenic acid content (Table 6), indicating an inverse relationship between these fatty acids (Zhong and Cheng 2017). As a major polyunsaturated fatty acid (PUFA) in *C. vulgaris* , linolenic acid contributes to membrane structure and stress signaling, suggesting that Se‐regulated tolerance mechanisms strongly influence fatty acid composition through metabolic pathway modulation (Freitas 2017).

ω‐3 and ω‐6 are two forms of essential polyunsaturated fatty acids for the proper functioning of the body. An imbalance created by excessive consumption of ω‐6 and inadequate intake of ω‐3 is an effective factor in numerous diseases in modern society. These fatty acids are essential for many bodily functions and need to be acquired through the diet, as the body cannot synthesize them. Therefore, a suitable ratio of ω‐6 to ω‐3 (n6/n3) is crucial for maintaining health and preventing obesity (A. P. A. P. Simopoulos 2016). The recommended ratio of ω‐6 to ω‐3 fatty acids in the human diet is generally considered to be around 1:1 to 4:1. This means that for optimal health, individuals should strive for a dietary balance where the consumption of ω‐3 fatty acids is equal to or up to four times that of ω‐6 fatty acids (A. A. Simopoulos 2003). According to the Food and Agriculture Organization (FAO) guidelines, the n‐6 to n‐3 fatty acid ratio should be maintained below 5 (WHO, F 2010). The findings indicated that the ratio of ω‐6 to ω‐3 fatty acids in *C. vulgaris* treated with 15 mg L^−1^ of Se and 50 mg L^−1^ of Fe was measured to be 1.75 and 1.78, respectively. This ratio corresponds with the recommended dietary standards.

Based on the fatty acid composition presented in Table 6, linoleic acid (C18:2) represented the predominant fatty acid in *C. vulgaris* across both the control and Se/Fe treatment groups, with palmitic acid (C16:0) and α‐linolenic acid (C18:3) ranking as the second and third most abundant, respectively. Linoleic and α‐linolenic acids, as essential polyunsaturated fatty acids, are important for biological functions and enhance the nutritional value of *Chlorella*. Thus, Se‐ and Fe‐enriched *C. vulgaris* show promising potential as a valuable dietary source of these essential fatty acids.

This study focused on assessing the effects of Se and Fe separately, with the aim of identifying the optimal concentration of each micronutrient for biofortifying *C. vulgaris* . Consequently, possible interactions between Se and Fe, particularly those involving shared metabolic pathways such as oxidative stress regulation and pigment synthesis, could not be examined. Further investigations combining Se and Fe treatments, along with other trace elements, are required to reveal potential synergistic or antagonistic effects and to extend the relevance of these results to microalgae‐based biofortification strategies.

## 5 Conclusion

In conclusion, our findings demonstrated that *C. vulgaris* effectively accumulated Se and Fe within its cells, reaching maximum levels of 81.54 mg kg^−1^ DW at 45 mg L^−1^ Se and 26798.51 mg kg^−1^ DW at 150 mg L^−1^ Fe, confirming its strong biofortification potential. However, the 15 mg L^−1^ of Se and 50 mg L^−1^ of Fe were identified as optimal concentrations for enriching *C. vulgaris* without compromising biomass quality, making them most suitable for functional food development. At these concentrations, the nutritional profile of *C. vulgaris* was significantly enhanced, as evidenced by increased production of certain photosynthetic pigments, including chlorophyll a, alongside elevated antioxidant activity, total phenolics, total flavonoids, and some key essential fatty acids. Based on the present experimental results and previously published reports, Se‐ and Fe‐biofortified *C. vulgaris* holds promising potential as a beneficial dietary supplement. Nevertheless, in vivo validation and clinical studies are necessary before formally establishing its application as a functional food or dietary supplement for human use.

## Author Contributions

**Akbar Hajizadeh Moghaddam:** data curation, investigation. **Mohammad Azadbakht:** project administration, supervision, conceptualization, writing – review and editing. **Mahbobeh Ghanbarzadeh:** data curation, investigation. **Masoumeh Rezaei Moshaei:** writing – original draft, formal analysis, software, visualization, data curation, investigation, methodology.

## Funding

The authors have nothing to report.

## Ethics Statement

This study does not involve any human or animal testing.

## Conflicts of Interest

The authors declare no conflicts of interest.

## Data Availability

The data that support the findings of this study are available from the corresponding author upon reasonable request.

## References

[fsn372230-bib-0001] Abdel‐Karim, O. H. , S. F. Gheda , G. A. Ismail , and A. M. Abo‐Shady . 2019. “Phytochemical Screening and Antioxidant Activity of *Chlorella vulgaris* .” Delta Journal of Science 41, no. 1: 79–91. 10.21608/djs.2020.139231.

[fsn372230-bib-0002] Abidizadegan, M. , J. Blomster , and E. Peltomaa . 2023. “Effect of Micronutrient Iron on Bioactive Compounds Isolated From Cryptophytes.” Frontiers in Plant Science 14: 1208724. 10.3389/fpls.2023.1208724. 37575946 PMC10413267

[fsn372230-bib-0003] Araie, H. , and Y. Shiraiwa . 2016. “Selenium in Algae.” Physiology of Microalgae: 281–288. 10.1007/978-3-319-24945-2_12.

[fsn372230-bib-0004] Arunakumara, K. , and X. Zhang . 2008. “Heavy Metal Bioaccumulation and Toxicity With Special Reference to Microalgae.” Journal of Ocean University of China 7: 60–64. 10.1007/s11802-008-0060-y.

[fsn372230-bib-0005] Ayed, H. B. A.‐B. , B. Taidi , H. Ayadi , D. Pareau , and M. Stambouli . 2015. “Effect of Magnesium Ion Concentration in Autotrophic Cultures of *Chlorella vulgaris* .” Algal Research 9: 291–296. 10.1016/j.algal.2015.03.021.

[fsn372230-bib-0006] Babaei, A. , K. Ranglová , J. R. Malapascua , and J. Masojídek . 2017. “The Synergistic Effect of Selenium (Selenite,–SeO 3 2−) Dose and Irradiance Intensity in Chlorella Cultures.” AMB Express 7: 1–14. 10.1186/s13568-017-0348-7. 28265976 PMC5339263

[fsn372230-bib-0007] Benzie, I. F. , and J. J. Strain . 1996. “The Ferric Reducing Ability of Plasma (FRAP) as a Measure of “Antioxidant Power”: The FRAP Assay.” Analytical Biochemistry 239, no. 1: 70–76. 10.1006/abio.1996.0292. 8660627

[fsn372230-bib-0008] Bito, T. , E. Okumura , M. Fujishima , and F. Watanabe . 2020. “Potential of Chlorella as a Dietary Supplement to Promote Human Health.” Nutrients 12, no. 9: 2524. 10.3390/nu12092524. 32825362 PMC7551956

[fsn372230-bib-0009] Blois, M. S. 1958. “Antioxidant Determinations by the Use of a Stable Free Radical.” Nature 181, no. 4617: 1199–1200. 10.1038/1811199a0.

[fsn372230-bib-0010] Bradford, M. M. 1976. “A Rapid and Sensitive Method for the Quantitation of Microgram Quantities of Protein Utilizing the Principle of Protein‐Dye Binding.” Analytical Biochemistry 72, no. 1–2: 248–254. 10.1016/0003-2697(76)90527-3. 942051

[fsn372230-bib-0011] Chomchan, R. , S. Siripongvutikorn , and P. Puttarak . 2017. “Selenium Bio‐Fortification: An Alternative to Improve Phytochemicals and Bioactivities of Plant Foods.” Functional Foods in Health and Disease 7, no. 4: 263–279. 10.31989/ffhd.v7i4.323.

[fsn372230-bib-0012] Dey, S. , and S. S. Raychaudhuri . 2023. “Selenium Biofortification Improves Bioactive Composition and Antioxidant Status in *Plantago ovata* Forsk, a Medicinal Plant.” Genes and Environment 45, no. 1: 38. 10.1186/s41021-023-00293-2. 38111072 PMC10729483

[fsn372230-bib-0013] Doušková, I. , J. Machát , D. Umysová , M. Vítová , J. Doucha , and V. Zachleder . 2007. “ *Scenedesmus quadricauda* ‐a Promising Microorganism for Selenium‐Enriched Algal Biomass Production.” https://is.muni.cz/osoba/9852.

[fsn372230-bib-0014] Duong, V. T. , S. R. Thomas‐Hall , and P. M. Schenk . 2015. “Growth and Lipid Accumulation of Microalgae From Fluctuating Brackish and Sea Water Locations in South East Queensland—Australia.” Frontiers in Plant Science 6: 359. 10.3389/fpls.2015.00359. 26042142 PMC4436584

[fsn372230-bib-0015] Ekelund, N. G. , and R. A. Danilov . 2001. “The Influence of Selenium on Photosynthesis and Light‐Enhanced Dark Respiration (LEDR) in the Flagellate *Euglena gracilis* After Exposure to Ultraviolet Radiation.” Aquatic Sciences 63: 457–465. 10.1007/s00027-001-8044-7. 10877069

[fsn372230-bib-0016] Freitas, H. R. 2017. “As a Source of Essential Fatty Acids and Micronutrients: A Brief Commentary.” Open Plant Science Journal 10: 92–99. 10.2174/1874294701710010092.

[fsn372230-bib-0017] Gąsecka, M. , M. Mleczek , M. Siwulski , P. Niedzielski , and L. Kozak . 2015. “The Effect of Selenium on Phenolics and Flavonoids in Selected Edible White Rot Fungi.” LWT‐ Food Science and Technology 63, no. 1: 726–731. 10.1016/j.lwt.2015.03.046.

[fsn372230-bib-0018] Ghanbarzadeh, M. , N. Moazami , S. Mirdamadi , M. H. Shahavi , M. R. Moshaei , and S. G. H. Kiadeh . 2024. “A Study on Some Phytochemicals in Arthrospira Platensis MGH‐1 Fortified With Calcium and Magnesium.” Phycologia 63, no. 4: 390–398. 10.1080/00318884.2024.2351207.

[fsn372230-bib-0019] Ghanbarzadeh, M. , N. Moazami , M. H. Shahavi , and S. Mirdamadi . 2022. “Study of Bioactive Compounds in Arthrospira Platensis MGH‐1 Fortified With Micronutrients of Iron, Zinc, and Manganese.” Journal of Applied Phycology 34, no. 5: 2449–2462. 10.1007/s10811-022-02797-w.

[fsn372230-bib-0020] Gojkovic, Ž. , I. Garbayo , J. L. G. Ariza , I. Márová , and C. Vílchez . 2015. “Selenium Bioaccumulation and Toxicity in Cultures of Green Microalgae.” Algal Research 7: 106–116. 10.1016/j.algal.2014.12.008.

[fsn372230-bib-0021] Gojkovic, Ž. , I. Garbayo‐Nores , V. Gómez‐Jacinto , et al. 2013. “Continuous Production of Selenomethionine‐Enriched *Chlorella sorokiniana* Biomass in a Photobioreactor.” Process Biochemistry 48, no. 8: 1235–1241. 10.1016/j.procbio.2013.06.013.

[fsn372230-bib-0022] Gojkovic, Ž. , C. Vílchez , R. Torronteras , et al. 2014. “Effect of Selenate on Viability and Selenomethionine Accumulation of *Chlorella sorokiniana* Grown in Batch Culture.” Scientific World Journal 2014, no. 1: 401265. 10.1155/2014/401265. 24688385 PMC3928859

[fsn372230-bib-0023] Guan, W. , H. Zhao , X. Lu , C. Wang , M. Yang , and F. Bai . 2011. “Quantitative Analysis of Fatty‐Acid‐Based Biofuels Produced by Wild‐Type and Genetically Engineered Cyanobacteria by Gas Chromatography–Mass Spectrometry.” Journal of Chromatography A 1218, no. 45: 8289–8293. 10.1016/j.chroma.2011.09.043. 21982444

[fsn372230-bib-0024] Haridas, S. , J. Ramaswamy , T. Natarajan , and P. Nedungadi . 2022. “Micronutrient Interventions Among Vulnerable Population Over a Decade: A Systematic Review on Indian Perspective.” Health Promotion Perspective 12, no. 2: 151–162. 10.34172/hpp.2022.19. PMC9508398 36276418

[fsn372230-bib-0025] Hasan, M. K. , Y. Cheng , M. K. Kanwar , X.‐Y. Chu , G. J. Ahammed , and Z.‐Y. Qi . 2017. “Responses of Plant Proteins to Heavy Metal Stress—A Review.” Frontiers in Plant Science 8: 1492. 10.3389/fpls.2017.01492. 28928754 PMC5591867

[fsn372230-bib-0026] HC, B. 1963. “Some Soil Algae From Enchanted Rock and Related Algal Species.” In Phycological Studies IV, 1–95. University of Texas Publ.

[fsn372230-bib-0027] Iolascon, A. , I. Andolfo , R. Russo , et al. 2024. “Recommendations for Diagnosis, Treatment, and Prevention of Iron Deficiency and Iron Deficiency Anemia.” Hema 8, no. 7: e108. 10.1002/hem3.108. PMC11247274 39011129

[fsn372230-bib-0028] Iriani, D. , S. Orasa , C. Nittaya , and H. Bustari . 2017. “Culturing of Chlorella sp. With Different of Iron (Fe3+) Concentration in Bold's Basal Medium for Healthy and Nutritious Cookies.” Applied Science and Technology 1, no. 1: 218–226.

[fsn372230-bib-0029] Iriani, D. , O. Suriyaphan , and N. Chaiyanate . 2011. “Effect of Iron Concentration on Growth, Protein Content and Total Phenolic Content of Chlorella sp. Cultured in Basal Medium.” Sains Malaysiana 40, no. 4: 353–358. http://www.ukm.my/jsm/index.html.

[fsn372230-bib-0030] Isani, G. , A. Niccolai , G. Andreani , et al. 2022. “Iron Speciation and Iron Binding Proteins in Arthrospira Platensis Grown in Media Containing Different Iron Concentrations.” International Journal of Molecular Sciences 23, no. 11: 6283. 10.3390/ijms23116283. 35682960 PMC9181241

[fsn372230-bib-0031] Islam, M. N. , F. Alsenani , and P. M. Schenk . 2017. “Microalgae as a Sustainable Source of Nutraceuticals.” Microbial Functional Foods and Nutraceuticals: 1–19. 10.1002/9781119048961.ch1.

[fsn372230-bib-0032] Ismaiel, M. M. , M. D. Piercey‐Normore , and C. Rampitsch . 2018. “Proteomic Analyses of the Cyanobacterium Arthrospira (Spirulina) Platensis Under Iron and Salinity Stress.” Environmental and Experimental Botany 147: 63–74. 10.1016/j.envexpbot.2017.11.013.

[fsn372230-bib-0033] Janczyk, P. , H. Franke , and W. Souffrant . 2007. “Nutritional Value of *Chlorella vulgaris* : Effects of Ultrasonication and Electroporation on Digestibility in Rats.” Animal Feed Science and Technology 132, no. 1–2: 163–169. 10.1016/j.anifeedsci.2006.03.007.

[fsn372230-bib-0034] Kumar, S. B. , S. R. Arnipalli , P. Mehta , S. Carrau , and O. Ziouzenkova . 2022. “Iron Deficiency Anemia: Efficacy and Limitations of Nutritional and Comprehensive Mitigation Strategies.” Nutrients 14, no. 14: 2976. 10.3390/nu14142976. 35889932 PMC9315959

[fsn372230-bib-0035] Lanza, M. G. D. B. , and A. R. Dos Reis . 2021. “Roles of Selenium in Mineral Plant Nutrition: ROS Scavenging Responses Against Abiotic Stresses.” Plant Physiology and Biochemistry 164: 27–43. 10.1016/j.plaphy.2021.04.026. 33962229

[fsn372230-bib-0036] Lattanzio, V. 2013. “Phenolic Compounds: Introduction 50.” Nat. Prod, 1543‐1580. 10.1007/978-3-642-22144-6_57.

[fsn372230-bib-0037] Leganés, F. , E. Sanchez‐Maeso , and E. Fernández‐Valiente . 1987. “Effect of Indoleacetic Acid on Growth and Dinitrogen Fixation in Cyanobacteria.” Plant and Cell Physiology 28, no. 3: 529–533. 10.1093/oxfordjournals.pcp.a077324.

[fsn372230-bib-0038] Lei, C. , Q. Ma , Q. Y. Tang , et al. 2014. “Sodium Selenite Regulates Phenolics Accumulation and Tuber Development of Purple Potatoes.” Scientia Horticulturae 165: 142–147. 10.1016/j.scienta.2013.10.024.

[fsn372230-bib-0039] Liang, T. , W. Yue , and Q. Li . 2010. “Comparison of the Phenolic Content and Antioxidant Activities of Apocynum Venetum L.(Luo‐Bu‐Ma) and Two of Its Alternative Species.” International Journal of Molecular Sciences 11, no. 11: 4452–4464. 10.3390/ijms11114452. 21151449 PMC3000093

[fsn372230-bib-0040] Liu, Z.‐Y. , G.‐C. Wang , and B.‐C. Zhou . 2008. “Effect of Iron on Growth and Lipid Accumulation in *Chlorella vulgaris* .” Bioresource Technology 99, no. 11: 4717–4722. 10.1016/j.biortech.2007.09.073. 17993270

[fsn372230-bib-0041] Lowe, N. M. 2021. “The Global Challenge of Hidden Hunger: Perspectives From the Field.” Proceedings of the Nutrition Society 80, no. 3: 283–289. 10.1017/S0029665121000902. 33896431

[fsn372230-bib-0042] Medicine, I. o . 2000. Dietary Reference Intakes for Vitamin C, Vitamin E, Selenium, and Carotenoids. National Academies Press. 25077263

[fsn372230-bib-0043] Medicine, I. o . 2001. Dietary Reference Intakes for Vitamin A, Vitamin K, Arsenic, Boron, Chromium, Copper, Iodine, Iron, Manganese, Molybdenum, Nickel, Silicon, Vanadium, and Zinc. National Academies Press. 10.1016/S0002-8223(01)00078-5. 25057538

[fsn372230-bib-0044] Nagata, M. , and I. Yamashita . 1992. “Simple Method for Simultaneous Determination of Chlorophyll and Carotenoids in Tomato Fruit.” Nippon Shokuhin Kogyo Gakkaishi 39, no. 10: 925–928. 10.3136/nskkk1962.39.925.

[fsn372230-bib-0045] Nićiforović, N. , V. Mihailović , P. Mašković , S. Solujić , A. Stojković , and D. P. Muratspahić . 2010. “Antioxidant Activity of Selected Plant Species; Potential New Sources of Natural Antioxidants.” Food and Chemical Toxicology 48, no. 11: 3125–3130. 10.1016/j.fct.2010.08.007. 20728497

[fsn372230-bib-0046] Pádrová, K. , J. Lukavský , L. Nedbalová , et al. 2015. “Trace Concentrations of Iron Nanoparticles Cause Overproduction of Biomass and Lipids During Cultivation of Cyanobacteria and Microalgae.” Journal of Applied Phycology 27: 1443–1451. 10.1007/s10811-014-0477-1.

[fsn372230-bib-0047] Palacios, I. , M. Lozano , C. Moro , et al. 2011. “Antioxidant Properties of Phenolic Compounds Occurring in Edible Mushrooms.” Food Chemistry 128, no. 3: 674–678. 10.1016/j.foodchem.2011.03.085.

[fsn372230-bib-0048] Pinto, E. , T. C. Sigaud‐kutner , M. A. Leitao , O. K. Okamoto , D. Morse , and P. Colepicolo . 2003. “Heavy Metal–Induced Oxidative Stress in Algae 1.” Journal of Phycology 39, no. 6: 1008–1018. 10.1111/j.0022-3646.2003.02-193.x.

[fsn372230-bib-0049] Porra, R. J. , W. A. Thompson , and P. E. Kriedemann . 1989. “Determination of Accurate Extinction Coefficients and Simultaneous Equations for Assaying Chlorophylls a and b Extracted With Four Different Solvents: Verification of the Concentration of Chlorophyll Standards by Atomic Absorption Spectroscopy.” Biochimica et Biophysica Acta (BBA)‐Bioenergetics 975, no. 3: 384–394. 10.1016/S0005-2728(89)80347-0.

[fsn372230-bib-0050] Rahaiee, S. , S. Ghanbari Hassan Kiadeh , M. Govahi , and M. Ghasemi . 2025. “Antioxidant, Anti‐Inflammatory, and Anticancer Activities of Ethanolic Chayote ( *Sechium edule* ) Fruit Extract: Phytochemical Insights and Therapeutic Implications.” Agrotechniques in Industrial Crops 6, no. 2: 135–142. 10.22126/atic.2026.12170.1221.

[fsn372230-bib-0051] Rastar, M. , S. Hosseini Shekarabi , M. Shamsaie Mehrgan , and S. Sabzi . 2018. “Effects of Iron and Zinc Concentrations on Growth Performance and Biochemical Composition of *Haematococcus pluvialis* : A Comparison Between Nanoparticles and Their Corresponding Metals Bulks.” Journal of Algal Biomass Utilization 9, no. 2: 59–67.

[fsn372230-bib-0052] Rizwan, M. , G. Mujtaba , and K. Lee . 2017. “Effects of Iron Sources on the Growth and Lipid/Carbohydrate Production of Marine Microalga *Dunaliella tertiolecta* .” Biotechnology and Bioprocess Engineering 22: 68–75. 10.1007/s12257-016-0628-0.

[fsn372230-bib-0053] Roosta, H. , A. Estaji , and F. Niknam . 2018. “Effect of Iron, Zinc and Manganese Shortage‐Induced Change on Photosynthetic Pigments, Some Osmoregulators and Chlorophyll Fluorescence Parameters in Lettuce.” Photosynthetica 56: 606–615. 10.1007/s11099-017-0696-1.

[fsn372230-bib-0054] Rusu, I. G. , R. Suharoschi , D. C. Vodnar , et al. 2020. “Iron Supplementation Influence on the Gut Microbiota and Probiotic Intake Effect in Iron Deficiency—A Literature‐Based Review.” Nutrients 12, no. 7: 1993. 10.3390/nu12071993. 32635533 PMC7400826

[fsn372230-bib-0055] Saffaryazdi, A. , M. Lahouti , A. Ganjeali , and H. Bayat . 2012. “Impact of Selenium Supplementation on Growth and Selenium Accumulation on Spinach ( *Spinacia oleracea* L.) Plants.” Notulae Scientia Biologicae 4, no. 4: 95–100. 10.15835/nsb448029.

[fsn372230-bib-0056] Safi, C. , B. Zebib , O. Merah , P.‐Y. Pontalier , and C. Vaca‐Garcia . 2014. “Morphology, Composition, Production, Processing and Applications of *Chlorella vulgaris* : A Review.” Renewable and Sustainable Energy Reviews 35: 265–278. 10.1016/j.rser.2014.04.007.

[fsn372230-bib-0057] Schiavon, M. , A. Ertani , S. Parrasia , and F. Dalla Vecchia . 2017. “Selenium Accumulation and Metabolism in Algae.” Aquatic Toxicology 189: 1–8. 10.1016/j.aquatox.2017.05.011. 28554051

[fsn372230-bib-0058] Shetty, A. , and G. Miller . 1966. “Influence of Iron Chlorosis on Pigment and Protein Metabolism in Leaves of *Nicotiana tabacum* L.” Plant Physiology 41, no. 3: 415–421. 10.1104/pp.41.3.415. 16656270 PMC1086358

[fsn372230-bib-0059] Siddiqui, S. A. , İ. Ucak , M. Afreen , et al. 2024. “Microalgae as a Potential Raw Material for Plant‐Based Seafood Alternatives: A Comprehensive Review.” Food Science & Nutrition 12, no. 11: 8559–8593. 10.1002/fsn3.4313. 39619996 PMC11606832

[fsn372230-bib-0060] Simopoulos, A. 2003. “The Importance of the Ratio of Omega‐6/Omega‐3 Essential Fatty Acids.” Alternative Medicine Review 8, no. 1: 83–84. 10.1016/S0753-3322(02)00253-6. 14579680

[fsn372230-bib-0061] Simopoulos, A. P. 2016. “An Increase in the Omega‐6/Omega‐3 Fatty Acid Ratio Increases the Risk for Obesity.” Nutrients 8, no. 3: 128. 10.3390/nu8030128. 26950145 PMC4808858

[fsn372230-bib-0062] Siva Kiran, R. , G. Madhu , and S. Satyanarayana . 2015. “Spirulina in Combating Protein Energy Malnutrition (PEM) and Protein Energy Wasting (PEW)‐A Review.” J. Nutr. Res 3, no. 1: 62–79.

[fsn372230-bib-0063] Sun, T. , S. Rao , X. Zhou , and L. Li . 2022. “Plant Carotenoids: Recent Advances and Future Perspectives.” Molecular Horticulture 2, no. 1: 3. 10.1186/s43897-022-00023-2. 37789426 PMC10515021

[fsn372230-bib-0064] Sun, X. , Y. Zhong , Z. Huang , and Y. Yang . 2014. “Selenium Accumulation in Unicellular Green Alga Chlorella Vulgaris and Its Effects on Antioxidant Enzymes and Content of Photosynthetic Pigments.” PLoS One 9, no. 11: e112270. 10.1371/journal.pone.0112270. 25375113 PMC4223018

[fsn372230-bib-0065] Takyar, M. B. T. , S. H. Khajavi , and R. Safari . 2019. “Evaluation of Antioxidant Properties of Chlorella Vulgaris and Spirulina Platensis and Their Application in Order to Extend the Shelf Life of Rainbow Trout ( *Oncorhynchus mykiss* ) Fillets During Refrigerated Storage.” LWT 100: 244–249. 10.1016/j.lwt.2018.10.079.

[fsn372230-bib-0066] Torres‐Tiji, Y. , F. J. Fields , and S. P. Mayfield . 2020. “Microalgae as a Future Food Source.” Biotechnology Advances 41: 107536. 10.1016/j.biotechadv.2020.107536. 32194145

[fsn372230-bib-0067] Trumbo, P. , A. A. Yates , S. Schlicker , and M. Poos . 2001. “Dietary Reference Intakes.” Journal of the American Dietetic Association 101, no. 3: 294. 10.1016/S0002-8223(01)00078-5. 11269606

[fsn372230-bib-0068] Vinceti, M. , G. Dennert , C. M. Crespi , et al. 2014. “Selenium for Preventing Cancer.” Cochrane Database of Systematic Reviews 3: 1–176. 10.1002/14651858.CD005195.pub3. PMC4441528 24683040

[fsn372230-bib-0069] Vítová, M. , K. Bišová , M. Hlavová , V. Zachleder , M. Rucki , and M. Čížková . 2011. “Glutathione Peroxidase Activity in the Selenium‐Treated Alga *Scenedesmus quadricauda* .” Aquatic Toxicology 102, no. 1–2: 87–94. 10.1016/j.aquatox.2011.01.003. 21371616

[fsn372230-bib-0070] Wang, C. A. , H. Onyeaka , T. Miri , and F. Soltani . 2024. “ *Chlorella vulgaris* As a Food Substitute: Applications and Benefits in the Food Industry.” Journal of Food Science 89, no. 12: 8231–8247. 10.1111/1750-3841.17529. 39556490 PMC11673457

[fsn372230-bib-0071] Wang, J. , and C. Chen . 2006. “Biosorption of Heavy Metals by *Saccharomyces cerevisiae* : A Review.” Biotechnology Advances 24, no. 5: 427–451. 10.1016/j.biotechadv.2006.03.001. 16737792

[fsn372230-bib-0072] Weng, H.‐X. , Y.‐C. Qin , X.‐W. Sun , H. Dong , and X.‐H. Chen . 2007. “Iron and Phosphorus Effects on the Growth of Cryptomonas sp.(Cryptophyceae) and Their Availability in Sediments From the Pearl River Estuary, China.” Estuarine, Coastal and Shelf Science 73, no. 3–4: 501–509. 10.1016/j.ecss.2007.02.002.

[fsn372230-bib-0073] WHO, F . 2010. Fats and Fatty Acids in Human Nutrition Report of an Expert Consultation. Food and Agriculture Organization of the United Nations 21812367. 21812367

[fsn372230-bib-0074] Yan, F. , K. Li , W. Xing , M. Dong , M. Yi , and H. Zhang . 2022. “Role of Iron‐Related Oxidative Stress and Mitochondrial Dysfunction in Cardiovascular Diseases.” Oxidative Medicine and Cellular Longevity 2022, no. 1: 5124553. 10.1155/2022/5124553. 36120592 PMC9473912

[fsn372230-bib-0075] Zdziebłowska, S. , J. Zajda , and L. Ruzik . 2024. “Microalgae Enriched in Selenium as a Good Source of Micronutrients.” Food Bioscience 59: 103908. 10.1016/j.fbio.2024.103908.

[fsn372230-bib-0076] Zhang, Z. , Y. Zhang , Y. Hua , G. Chen , P. Fu , and J. Liu . 2024. “Heterotrophic Selenium Incorporation Into *Chlorella vulgaris* K‐01: Selenium Tolerance, Assimilation, and Removal Through Microalgal Cells.” Food 13, no. 3: 405. 10.3390/foods13030405. PMC10855183 38338539

[fsn372230-bib-0077] Zhao, Y. , X. Song , X. Cao , Y. Wang , Z. Si , and Y. Chen . 2019. “Toxic Effect and Bioaccumulation of Selenium in Green Alga *Chlorella pyrenoidosa* .” Journal of Applied Phycology 31: 1733–1742. 10.1007/s10811-018-1711-z.

[fsn372230-bib-0078] Zhong, Y. , and J. J. Cheng . 2017. “Effects of Selenite on Unicellular Green Microalga *Chlorella pyrenoidosa* : Bioaccumulation of Selenium, Enhancement of Photosynthetic Pigments, and Amino Acid Production.” Journal of Agricultural and Food Chemistry 65, no. 50: 10875–10883. 10.1021/acs.jafc.7b04246. 29179543

